# Hydrocarbon Degradation in Caspian Sea Sediment Cores Subjected to Simulated Petroleum Seepage in a Newly Designed Sediment-Oil-Flow-Through System

**DOI:** 10.3389/fmicb.2017.00763

**Published:** 2017-04-28

**Authors:** Sonakshi Mishra, Peggy Wefers, Mark Schmidt, Katrin Knittel, Martin Krüger, Marion H. Stagars, Tina Treude

**Affiliations:** ^1^GEOMAR Helmholtz Center for Ocean Research KielKiel, Germany; ^2^Max Planck Institute for Marine MicrobiologyBremen, Germany; ^3^Federal Institute for Geosciences and Natural ResourcesHannover, Germany; ^4^Department of Earth, Planetary and Space Sciences, University of California, Los Angeles, Los AngelesCA, USA; ^5^Department of Atmospheric and Oceanic Sciences, University of California, Los Angeles, Los AngelesCA, USA

**Keywords:** crude oil, sulfate reduction, methanogenesis, oxygen consumption, *n*-alkanes, sulfide, methane, porosity

## Abstract

The microbial community response to petroleum seepage was investigated in a whole round sediment core (16 cm length) collected nearby natural hydrocarbon seepage structures in the Caspian Sea, using a newly developed Sediment-Oil-Flow-Through (SOFT) system. Distinct redox zones established and migrated vertically in the core during the 190 days-long simulated petroleum seepage. Methanogenic petroleum degradation was indicated by an increase in methane concentration from 8 μM in an untreated core compared to 2300 μM in the lower sulfate-free zone of the SOFT core at the end of the experiment, accompanied by a respective decrease in the δ^13^C signal of methane from -33.7 to -49.5‰. The involvement of methanogens in petroleum degradation was further confirmed by methane production in enrichment cultures from SOFT sediment after the addition of hexadecane, methylnapthalene, toluene, and ethylbenzene. Petroleum degradation coupled to sulfate reduction was indicated by the increase of integrated sulfate reduction rates from 2.8 SO_4_^2-^m^-2^ day^-1^ in untreated cores to 5.7 mmol SO_4_^2-^m^-2^ day^-1^ in the SOFT core at the end of the experiment, accompanied by a respective accumulation of sulfide from 30 to 447 μM. Volatile hydrocarbons (C2–C6 *n*-alkanes) passed through the methanogenic zone mostly unchanged and were depleted within the sulfate-reducing zone. The amount of heavier *n*-alkanes (C10–C38) decreased step-wise toward the top of the sediment core and a preferential degradation of shorter (<C14) and longer chain *n*-alkanes (>C30) was seen during the seepage. This study illustrates, to the best of our knowledge, for the first time the development of methanogenic petroleum degradation and the succession of benthic microbial processes during petroleum passage in a whole round sediment core.

## Introduction

**FIGURE 1:**
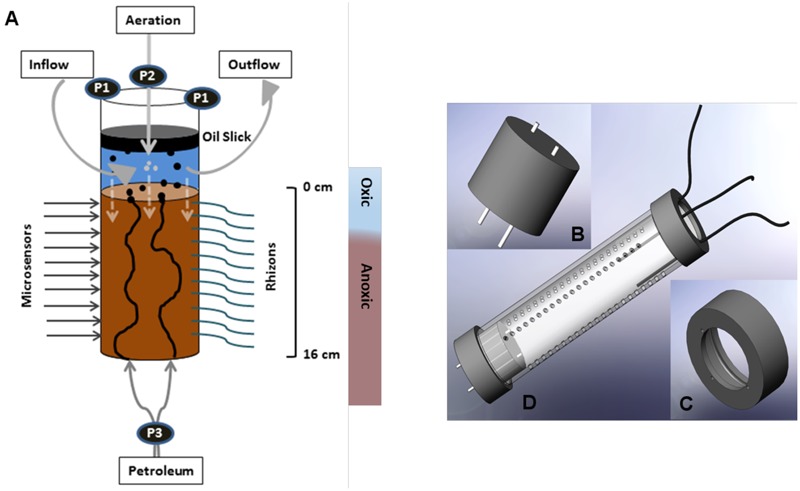
**(A)** Schematic diagram of the SOFT system for the simulation of petroleum seepage through whole round sediment cores. Artificial seawater was ventilated through the supernatant (P1, pump rate 25 μL min^-1^) and aerated with an air pump (P2). Petroleum was supplied in by a pump (P3) at 3.5 μL min^-1^ through two integrated channels within the bottom sealing. Vertically aligned rhizons (2.5 mm diameter) were permanently fixed for frequent extraction of porewater. Silicon-sealed holes (4 mm diameter) on the opposite side were used for microsensor measurements. From the oxic supernatant electron acceptors (e.g., O_2_, sulfate) entered the sediment by diffusion (dashed white arrows). **(B–D)** Individual parts of the SOFT system (technical drawing). **(B)** Rubber stopper with two integrated steel channels **(C)** Upper cap with three small holes to guide tubing for aeration and seawater inflow/outflow **(D)** Iso-versinic tubing assembly of individual parts with the core liner.

**FIGURE 2:**
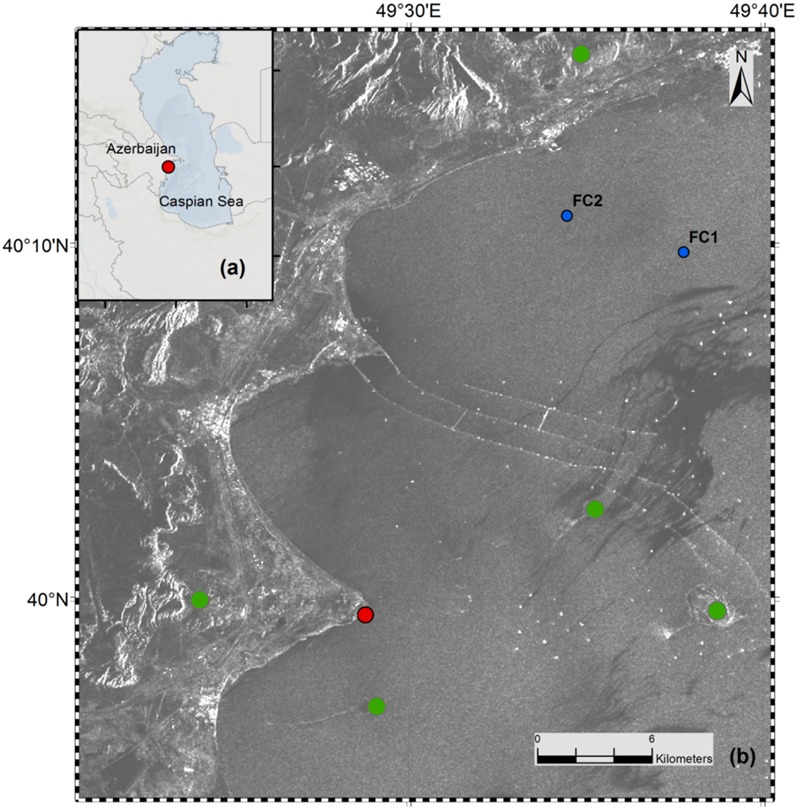
**(a)** Map of Azerbaijan and the Caspian Sea. **(b)** Geographical map showing the push-core sampling area (red dot). Characteristic features like on- and offshore mud volcanoes (green dots), abandoned offshore wells and infrastructures (white spots and lines in the image), and a central oil slick area (dark gray area in the image) are indicated. FC1 and FC2 are nearby sites where geochemical analyzes were done by Jost (2014). Map was produced by using ArcGIS 10.2, and is based on a regional SAR image taken in 2004 by ENVISAT (© ESA (2010), European Space Agency, ESA).

Geothermal action on kerogen in fine-grained sedimentary rocks leads to the formation of petroleum over geological timescales. Petroleum then sometimes migrates from its source rock and accumulates, forming reservoirs, when overlaying impermeable rocks block its upward movement. From these reservoirs, petroleum may seep to the sediment/soil surface through faults and cracks driven by buoyancy, capillary pressure and hydrodynamic gradients (Tissot and Welte, 1984). The two principal processes, through which petroleum enters the marine environment, are either naturally through seepage (for example, Allen et al., 1970) or via anthropogenic accidents like oil spills (Water, 2011). It is estimated that 600 metric tons of oil enter the ocean each year via natural seeps accounting for 47% of the total petroleum input to the marine environment (Kvenvolden and Cooper, 2003). Here, petroleum is subjected to weathering by physical, chemical and biological processes (Wardlaw et al., 2008) and microbial degradation is the most important degradation process involved (Das and Chandran, 2011 and references therein). Unlike marine oil spills, where petroleum enters through the oxygenated water column undergoing powerful breakdown by aerobic respiration (Head et al., 2006), petroleum in natural seeps enters from the anoxic and energetically lower end of the redox cascade. Hence, a different succession of microbial steps is expected in seeps compared to spills. Many studies have focused on the microbial degradation of spilled oil in the oceans’ water column (for example, Delvigne and Sweeney, 1988; Atlas, 1991; Prince et al., 2003; Jiménez et al., 2006; Prince et al., 2013), but relatively few studies investigated the microbial degradation of petroleum in hydrocarbon seeps (for example, Wenger and Isaksen, 2002; Wardlaw et al., 2008; Orcutt et al., 2010). Despite the increase in the number of studies on anaerobic degradation of hydrocarbons, there is still a lack of understanding how hydrocarbon-degraders act as a community in the environment and how petroleum is successively degraded under anoxic conditions (Head et al., 2006; Widdel et al., 2010). So far, selective utilization of hydrocarbons has been classically studied in enrichment cultures and isolates (for example, Ehrenreich et al., 2000; Rockne and Chee-Sanford, 2000; Cravo-Laureau et al., 2007; Kniemeyer et al., 2007). However, the use of batch cultures is insufficient to know the fate of petroleum in a natural ecosystem (Horowitz and Atlas, 1977). Because it is impossible to mimic all environmental determinants in the laboratory, Horowitz and Atlas suggested that the best chance to predict the fate of petroleum in a natural ecosystem is through chemostats, which maintain a constant influx and efflux of nutrients and products, respectively. There are few studies in the literature that are based on continuous flow-through systems to study petroleum hydrocarbon degradation (Bertrand et al., 1986) and oil spill scenarios (Horowitz and Atlas, 1977), but none on petroleum seepage in marine sediments. Investigations of hydrocarbon seeps often capture only snapshots of biogeochemical features (for example, Bauer et al., 1988; Wenger and Isaksen, 2002; Orcutt et al., 2008; Wardlaw et al., 2008) and are unable to follow the succession of processes related to petroleum seepage through natural sediment. In the present study, we developed a Sediment-Oil-Flow-Through (SOFT) system, modified from the Sediment-Flow-Through (SLOT) system (Steeb et al., 2014). While the SLOT system simulates a natural methane seep in whole round sediment cores, the SOFT system simulates petroleum-seep-like conditions (**Figure 1**). The system enables the monitoring of biogeochemical alterations in the sediment core during petroleum seepage over time. For our study, we collected sediment cores from the Caspian Sea (**Figure 2**), which is one of the oldest petroleum-producing regions in the world with enormous oil and gas reserves (Effimoff, 2000). Offshore drilling and land-based activities such as oil refineries, petrochemical plants, pipeline constructions have led to pollution and contamination of the Caspian Sea (Karpinsky, 1992; Dumont, 1995, 1998; Abilov et al., 1999). Moreover, natural hydrocarbon transport from greater depth to soil/sediment surface (e.g., by mud volcanism) is described for the South Caspian Basin (Katz et al., 2000; Akper, 2012). As the Caspian Sea is an enclosed basin, pollutants discharged into it accumulate and are partly trapped, e.g., in surface sediment. However, so far only a few studies have focused on the microbial community and petroleum degradation in sediments from the Caspian Sea (Hassanshahian et al., 2012; Hassanshahian, 2014; Mahmoudi et al., 2015).

The aim of the present study was to investigate the development of biogeochemical gradients related to microbial petroleum degradation and the successive consumption of hydrocarbons in Caspian Sea sediment during a simulated seepage through a whole round sediment core. We hypothesize that petroleum seepage through the Caspian Sea sediment will affect the vertical (i) zonation of redox processes, (ii) distribution and activity of petroleum-degrading microbial communities, and (iii) composition of seeping petroleum. We used the SOFT system to identify the above processes as a function of petroleum seepage to address the aforementioned hypotheses. This study presents detailed datasets on the successive biogeochemical response of the sediment to petroleum seepage and the alteration of the petroleum hydrocarbons. The biogeochemical data obtained here, were then correlated with microbial community analysis in a study by Stagars et al. (2017).

## Materials and Methods

### Study Site and Field Sampling

The Caspian Sea is the largest continental water body and the rivers Volga, Kura, and Ural are the three biggest contributors of its inflow and nutrients (Dumont, 1998). It has an area exceeding 390,000 km^2^ with a water volume of around 78,000 km^3^ (Kosarev, 2005). The salinity of the Caspian Sea is around 12 psu and the relative concentrations of SO_4_^2-^ (∼31 mM), Ca^2+^ (∼8 mM) and Mg^2+^ (∼29 mM) are higher than in average seawater due to the inflowing rivers Volga and Kura (Millero and Chetirkin, 1980; concentrations from Peeters et al., 1999). The sampling site was located in the South Caspian Sea basin at a coastal area near Baku, Azerbaijan (N 39 59.548, E 49 28.775). The site was chosen due to presence of nearby natural hydrocarbon seepage structures like off and on shore mud volcanoes (**Figure 2**). The water depth at the sampling site was around 60 cm. Sediment cores were collected in November 2012 by directly walking into the water and pushing core liners into the sediment by hand. Three large replicate sediment push cores (polycarbonate liner, 30 cm long, 6 cm inner diameter) were collected (**Figure 1**) for sediment analyzes and the SOFT experiment. Additionally, two replicate mini push cores (polycarbonate liner, 30 cm long, 2.6 cm inner diameter) were collected for determination of sulfate reduction rates (SRRs). The sediment cores were sealed air-free (filled with seawater to the brim) with rubber stoppers. The cores were stored at the Geological Institute of Azerbaijan in the dark at ∼10°C until they were shipped to Kiel in February 2013. Two of the large push cores were used for immediate sediment and porewater analyzes, respectively, upon arrival in Kiel. The cores were 18 cm and 16 cm long, respectively. The two replicate mini push cores (12 and 16 cm long) were used for SRR determination immediately upon arrival in Kiel. One large push core (16 cm long) was selected for the SOFT experiment. The SOFT core was stored at 0°C in the dark to reduce microbial activity until the SOFT system was established in May 2013. In the following, cores sampled upon arrival in Kiel will be called “untreated cores” in opposite to the SOFT core, which was used in the simulated petroleum seepage experiment. Untreated cores are here defined as sediment cores that were not subjected to petroleum addition in this study and hence, would serve as a reference for sediment conditions prior to the applied petroleum seepage. It should be mentioned, however, that due to the delay between the sampling of the untreated cores and the start of the SOFT experiment (3 months), SOFT-core sediment might have been subject to alteration during storage; however, we expected these changes to be small given the low storage temperature (0°C) and the lack of light to support photosynthesis. In order to test if the storage time had major effects on bacterial diversity in the sediment cores, a comparison of bacterial diversity was done between an untreated core that was sampled upon arrival in Kiel and an untreated core that was stored for 3 years at 0°C in the dark. No significant difference between the cores was observed (Stagars et al., 2017).

### Setup of the SOFT System

A SOFT System was developed to simulate petroleum seepage in whole round sediment cores by pumping petroleum through the bottom and providing diffusive supply of dissolved electron acceptors such as oxygen, nitrate, and sulfate via oxic artificial seawater from the top (**Figure 1**). The SOFT system was modified after the SLOT system established by Steeb et al. (2014), which simulates methane seepage (see Supplement 1 for a detailed comparison). A whole round sediment core was collected with a gas-tight polycarbonate core liner (**Figure 2** and Supplement 1). The upper end of the core liner was provided with a PVC cap with a central opening (diameter 4 cm) and three small openings (diameter 3.5 mm). The three small openings were used to feed through tubing (Iso-versinic, LLG; inner diameter 1 mm and outer diameter 3 mm) from an air pump and a seawater reservoir (inflowing artificial seawater to the SOFT core), and into a wastewater reservoir (outflowing seawater from the SOFT core). The cap was semi open and wrapped with permeable laboratory film (Parafilm, Pechiney Plastic Packaging), to allow gas exchange with the atmosphere. Aeration by air pump in the supernatant water was applied to facilitate a natural redox zonation (from oxic to anoxic) in the sediment core. Care was taken that the air flow was not too strong to avoid disturbance of the sediment surface layer. The bottom part of the core was kept anoxic by sealing the end of the core liner with a gas-tight rubber stopper (**Figure 1**). Two metal tubes cut from biopsy needle holder (O-MAX T Knochenmark-Biospie-Set; outer diameter 3 mm, inner diameter 1.9 mm) were integrated in the rubber stopper as crude-oil inlets. All tubing connections (sediment core, crude oil reservoir, seawater reservoir, collection bottle and air pump) were established with gas tight and autoclavable Iso-versinic tubes (LLG), polypropylene tube connectors and fast couplers. Crude oil was pumped into the sediment core at ∼3.5 μl min^-1^ with peristaltic pumps (Medorex, TL/10E, min/max pump volume 0.1 μL min^-1^/400 μl min^-1^) using Santropen tubes (Medorex; autoclavable, high flexible, very resistant against corrosive media; inner diameter 0.5 mm, outer diameter 1.6 mm). Petroleum pumping was switched on and off at frequent intervals (2–3 days of no-flow vs. 2–3 days of oil-flow) to imitate natural variability of petroleum migration and to offer organisms sufficient time for degradation in their microhabitat. A layer of petroleum (oil slick) formed at the water–air interface from the petroleum that seeped out of the sediment core. The oil slick was periodically removed with sterile syringes to avoid overflow. Light, live crude oil, i.e., oil featuring low viscosity and specific gravity and containing dissolved gas in solution, was provided by Dea Deutsche Erdoel AG and originated from the North Sea (Mittelplate; sampled in February 2013). The crude oil was not directly analyzed for the presence of bacteria. A pre-study was conducted, in which sediment (from the Eckernförde Bay, Baltic Sea) was mixed with different percentages of oil (from 0.6 to 50%). Thereby, an increasing percentage of dead cells, reaching almost 100% in the 50:50 mixture, was detected (LIVE/DEAD^®^ *Bac*Light^TM^ Bacterial Viability Kit; K. Laufer, unpublished data). We therefore assume that the undiluted crude oil was unable to sustain a viable microbial community. Artifical seawater, adapted to salt/sulfate concentrations found at the study site (further details below), was continously supplied to the supernatant (ca. 5 cm fill height) with persitaltic pumps through an inlet tube at a flow rate of 25 μl min^-1^. Simultaneously, an outlet tube was placed at a level higher than the artificial seawater inlet tube, which removed the liquid at 25 μl min^-1^ to maintain a constant level of supernatant. Cotton plugs were applied to the artificial seawater reservoir to maintain sterile and oxic conditions. The outlet tube connecting the artificial seawater reservoir with the SOFT liner was integrated into the cotton plug. The artificial seawater reservoir bottle, the cotton plug, and the inlet were autoclaved at 120°C for 65 min prior to usage. Artificial seawater was prepared by mixing 12 g of sea salts (Sigma–Aldrich, product number S9883) in 1000 ml of sterile deionized water to achieve a salinity of 12 psu, according to the salinity found at the study site (see methods of porewater analyzes below). No specific nutrients or vitamins were added to avoid unnatural enrichments of microorganisms. Additional sulfate was added in the form of magnesium sulfate (5.7 g of MgSO_4_^.^7H_2_O) to obtain an end concentration of 30 mmol L^-1^ close to the sulfate concentrations found at the study site (see results). The 2.5 mL of 10 mM KNO_3_ was added to obtain an end concentration of 25 μM NO_3_^-^ in the artificial seawater. The entire SOFT experiment was carried out in the dark in an incubator at 16°C. The temperature represents the average temperature of Caspian Sea surface water in Baku in November 2012, i.e., during our sampling campaign (source^1^).

### Microelectrode Measurements

Microelectrode measurements for dissolved oxygen and sulfide were done exclusively for the SOFT core, periodically (every 30 to 40 days) after the start of the SOFT experiment. Total dissolved sulfide was measured with a needle H_2_S microelectrode (Unisense, Denmark; H_2_S–N, tip diameter 0.8 mm) according to Steeb et al. (2014). The sensor was calibrated by six different concentrations of Na_2_S standard solution (0, 100, 200, 500, 1000, 2000 μmol l^-1^). The standards were prepared with oxygen-free citric acid-phosphate buffer, 10% v/v TiCl and set to pH 7.5 as this value represented the relatively consistent average pH of the sediment core (data not shown). Hence, it has to be kept in mind that total sulfide data were not corrected for individual pH data points. The microsensor calibration was done prior to measurements using the calibration software offered by Unisense (SensorTrace PRO), which provided signals in millivolt and the corresponding concentration for each data point. The data were corrected for the shift in the electronic signal at the end of the measurement.

**FIGURE 3:**
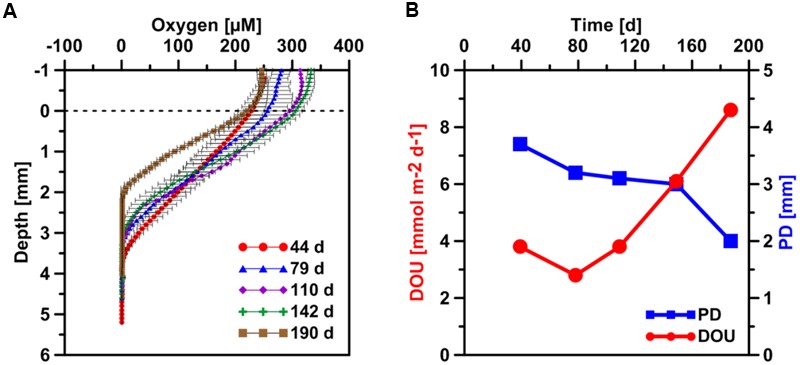
**(A)** Temporal development of sediment microprofiles of dissolved oxygen after the start of the SOFT experiment. Values are mean of three separate vertical profiles but with different horizontal positions (±SD, *n* = 3). The dashed horizontal line represents the sediment-water interface. **(B)** Temporal development of the oxygen penetration depth (PD) and the diffusive oxygen uptake (DOU) generated from the mean oxygen profiles.

Oxygen was measured with miniaturized Clark-type glass microelectrode (Unisense, Denmark; OX-100, tip diameter 100 μm). As the overlaying seawater in the SOFT core was constantly bubbled with air and sealed with Parafilm (Pechiney Plastic Packaging, Menasha) we assumed 100% oxygen saturation. Therefore, a two point calibration was done using the overlaying water as 100% atmospheric oxygen and the lowest signal in the sediment as the zero reading (0% oxygen) according to Sommer et al. (2010). Vertical profiling over 5 cm depth was done with a step size of 100 μm measuring period of 3 s and waiting period of 15 s. The vertical profiling was repeated three times to get the standard deviation shown as error bars (**Figure 3**). Oxygen penetration depth (PD) and the diffusive oxygen uptake (DOU) for the SOFT core were calculated from mean microsensor profiles according to Glud et al. (1994).

### Porewater Sampling

Rhizons (Rhizosphere, CSS-F, length 5 cm, diameter 2.5 mm, pore size 0.2 μm) were used for sediment porewater extractions from the untreated and the SOFT core through a vertical sampling-hole line of the core liners (for details, see Steeb et al., 2014). Around 1.5 to 2 ml porewater was extracted per rhizon every 1 to 2 cm intervals. Out of the 2 ml extracted porewater, around 0.05 to 0.1 ml of the porewater was used for immediate measurement of total alkalinity. The rest of the porewater was stored in 2 ml plastic cyro-vials at -20°C, and was later used for the analyzes of sulfate.

### Porewater Analyzes

Porewater concentration of sulfate was determined by ion-chromatography (for details, see Steeb et al., 2014). Total dissolved sulfide was determined photometrically at 670 nm after (Cline, 1969). Total alkalinity was determined by titrating 50 or 100 μl sample by 0.01 M HCl titrosol solution according to Ivanenkov and Lyakhin (1978) with an electric burette (876, Dosimat plus, metrohm) (for details, see Steeb et al., 2014). In addition to porewater analyzes, the salinity of the overlaying seawater of the untreated sediment core was tested with a refractometer (Master S-Millα, ATAGO, Tokyo, Japan).

### Sediment Core Slicing and Sampling

Sediment samples of both the untreated and the SOFT core were collected from sediment cores by slicing for subsequent solid phase analyzes.

#### Untreated Core

One of the large push cores was sampled upon arrival in Kiel. For slicing, the bottom stopper was removed and the core liner was placed on an extruder (diameter ∼5.8 cm). The sediment core was sliced vertically from top to bottom every 1 (0–5 cm) to 2 cm (5 cm until end of core). Sediment slices were then subsampled for the analyzes of methane (C1) and its δ^13^C-methane isotopic signature, porosity, total carbon (C), nitrogen (N), 4′,6′-diamidino-2-phylindole (DAPI) staining, Catalyzed Reporter Deposition Fluorescence In Situ Hybridization (CARD – FISH) (see Stagars et al., 2017), and 16S rRNA phylogenetic studies (see Stagars et al., 2017).

#### SOFT Core

The SOFT core was sampled at the end of the experiment, i.e., after 190 days of simulated petroleum seepage. The sediment core was sliced vertically from top to bottom every 1 (0–3 cm) to 2 cm (3 cm until end of core). During removal of the supernatant with a syringe, an oil slick settled on the sediment surface. The top (0–1 cm) sediment layer of the core is therefore considered non-representative for some of the sediment parameters (e.g., *n*-alkane alteration) due to potential mixing with petroleum hydrocarbons from the overlying oil slick. In addition to the parameters mentioned for the untreated core above, the SOFT core was also subsampled for the analyzes of volatile (C2–C6) *n*-alkanes, heavier (C10–C38) *n*-alkanes, SRRs, and enrichment culturing of methanogens and sulfate reducers.

### Sediment Analyzes: Volatile *n*-Alkanes (C1–C6) and Their Carbon Isotopic Signature

Dissolved volatile hydrocarbons (C1–C6) in sediment subsamples were determined by the headspace technique. Two milliliters of sediment and 5 ml of 2.5% (w/w) NaOH solution were equilibrated in a septum-sealed 13 ml headspace glass vial at room temperature (Sommer et al., 2009). Final concentrations of C1–C6 *n*-alkanes are presented per volume porewater after porosity correction. For the analyzes of volatile hydrocarbons in the original crude oil, 2 ml of the oil and 5 ml of 2.5% (w/w) NaOH solution were equilibrated in a septum-sealed 13 ml headspace glass vial at room temperature. It should be noted, however, that we cannot completely exclude losses of volatiles from the original crude oil as it was analyzed after the SOFT experiment, during which it was kept stored at room temperature in its original sealed container. Hydrocarbon (C1–C6) composition of the headspace gas was then determined with a “Thermo Trace ultra” (Thermo Scientific) gas chromatograph equipped with flame ionization detector (carrier gas: helium 5.0; capillary column: RT Alumina Bond-KCl, column length: 50 m; column diameter: 0.53 μm). Precision of ±1–3% was achieved in comparison to standard hydrocarbon mixtures. Stable carbon isotope ratios of methane (C1) were determined by continuous flow GC combustion – Isotope Ratio Mass Spectrometer combination. Hydrocarbons were separated in a Thermo Trace GC (carrier gas: He; packed column: ShinCarbon, 1.5 m). The subsequent conversion of methane to carbon dioxide was conducted in a Ni/Pt combustion furnace at 1150°C. The ^13^C/^12^C-ratios of the produced CO_2_ were determined by a Thermo MAT253 isotope ratio mass spectrometer. All isotope ratios are reported in the δ-notation with respect to Vienna Pee Dee Belemnite (VPDB). Analytical precision of the reported isotopic composition was ±0.3‰. A detection limit of 10 ppmV methane (1 ml syringe injection) had been achieved by using the movable capillary device “DIPcon^®^” for the reference CO_2_ inlet instead of the original fixed one of Thermo Scientific^TM^ (Cordt, 2012).

### Sediment Analyzes: C10–C38 *n*-Alkanes

Sediment subsamples were collected in aluminum foil and frozen at -20°C until analyzes. Prior to extraction, the sediment samples were allowed to thaw at 4°C. Samples were extracted with an Accelerated Solvent Extraction (Dionex ASE 150, Thermo Scientific) and measured in gas chromatograph-mass spectrometer (GC-MS) (Shimadzu, GCMS-QP2010 with auto injector AOC-20i) for *n*-alkanes. Around 4 g of sediment (mixed with inert diatomaceous earth) was extracted with dichloromethane and acetone (80:20) at 100°C with the ASE (Application note 338, Dionex). The extract was dried over sodium sulfate and was passed through a glass chromatographic column (Eydam, length 25 cm, inner diameter 1 cm). The chromatographic column was filled with 1 g of silica gel (Roth, 230–400 μm mesh size, preheated at 450°C for 4 h and activated with 8% v/w with deionized water) and contained silanized glass wool at the outlet end. The column was then washed portion wise with 20 ml of *n*-hexane and the resulting extract was collected and dried in a conical flask. The conical flask was then washed with 2 ml of *n*-hexane and this final solution was measured with GC-MS (1:10 or 1:20 dilution depending on the sample). Before the extraction process, deuterated tetracosane (C_24_D_50_) was used as an internal standard in the extraction cell. The 100 μL of 2000 ng μL^-1^ C_24_D_50_ was added to the extraction cell to achieve an end concentration of 20 ng μl^-1^ C_24_D_50_ in the final extract. A solution of 20 ng μl^-1^ C_24_D_50_ in *n*-hexane was measured separately to produce a reference peak area on the chromatogram. The ratio of the two chromatogram peak areas (sample extract and reference) of the internal standard was used to calculate the recovery of individual *n*-alkanes in our samples. The GC-MS had a capillary column (ZB-1HT Inferno, length 30 m, thickness 0.25 μm, diameter 0.25 mm). Helium Alphagaz-1(Air Liquide) was used as the carrier gas with a flow rate of 0.8 ml min^-1^. The samples were measured in scan mode with the mass-to-charge ratio (m/z) range of 43 to 85. The original crude oil was extracted in the same procedure by mixing it with inert diatomaceous earth prior extraction by ASE. To get a precision of the methodology, the original crude oil was extracted four times (from the first step of extraction to the final measurement step). A method precision range for each *n*-alkane in the original crude oil is provided in Supplement 2. Instrument precision for each *n*-alkane determined by repeated measurements of 1 ng μl^-1^ standard mix is provided in Supplement 2.

### Sediment Analyzes: Porosity, Carbon, and Nitrogen

Porosity was determined by weighing the wet and freeze-dried weights of sediment from both the untreated and the SOFT cores. Porosity was then calculated from the water content assuming a dry solid density of 2.63 g cm^-3^. As the bulk volume of petroleum was not removed by the freeze-drying process, porosity values might appear lower than they actually were in samples that contained oil. Porosity samples were subsampled for analyzes of total carbon (TC) and nitrogen (TN) and analyzed by CARLO ERBA Elemental Analyzer (NA 1500) (Steeb et al., 2014). Total organic carbon (TOC) was then determined by the difference in carbon content after removing the total inorganic carbon (TIC) through acidification (addition of 0.25 N HCl). Measurements were done in duplicates.

### Sediment Analyzes: Sulfate Reduction Rates

#### Untreated Cores

Upon arrival in Kiel, 6 μl of the carrier-free ^35^SO_4_^2-^ radiotracer (dissolved in water, 200 kBq, specific activity 37 TBq mmol^-1^) was injected into the two untreated replicate mini push cores in 1-cm depth intervals according to the whole-core injection method (Jørgensen, 1978). The mini cores were incubated for 6.5 h at 16°C. After incubation, bacterial activity was stopped by slicing the push core in 1 cm intervals and transferring each sediment layer into 50 ml plastic centrifuge tubes filled with 20 ml zinc acetate (20% w/w).

#### SOFT Core

Three to 4 ml sediment were sampled every 2 cm into 5 ml glass tubes and immediately sealed with butyl rubber stoppers (Treude et al., 2005). The tubes were injected with 6 μl carrier-free ^35^SO_4_^2-^ radiotracer (dissolved in water, 200 kBq, specific activity 37 TBq mmol^-1^) and incubated at 16°C for 12 h. Incubation was ended by transferring the sediment into 50 ml plastic centrifuge tubes filled with 20 ml zinc acetate (20% w/w).

Controls for all incubations (untreated and SOFT core) were prepared by adding tracer to killed samples. Centrifuge vials were stored at -20°C until rate determination by the cold chromium distillation procedure according to Kallmeyer et al. (2004).

### Sediment Analyzes: DAPI Staining

Sediment samples were fixed in 3% formaldehyde for 3 h at 4°C, washed with 1x phosphate-buffered saline (PBS) and stored in ethanol-PBS (1:1) at -20°C. Samples were diluted, four times ultrasonicated on ice at 20% intensity, 20 cycles, 30 s (Bandelin Sonopuls HD200). An aliquot was filtered on a 0.22 μm pore size polycarbonate filter. Filter sections were embedded in Citifluor:Vectashield (4:1) mounting medium containing 50 μg ml^-1^ DAPI. Microscopy was done with a Nikon eclipse 50i epifluorescence microscope.

### Enrichment Culturing of Methanogenic Hydrocarbon Degraders

Anaerobic incubations with sediment samples from the SOFT core were set up in an anaerobic chamber to determine methanogenic rates and the potential of indigenous microorganisms to degrade selected hydrocarbons. One gram of sediment from both the sulfate-reducing and methanogenic zone of the SOFT core was each transferred into two separate autoclaved 50-ml glass bottles containing 20 ml of sulfate-free seawater medium (Widdel and Bak, 1992). The salinity of the medium was adapted to the respective original seawater conditions (12 psu) by adding varying amounts of NaCl (Merck, CAS-No: 7647-14-5). The glass vials were sealed with sterile butyl rubber stoppers and aluminum crimp caps. All tubes were flushed with N_2_ to remove traces of H_2_ from the anaerobic chamber. Zero and 20 mM sulfate were added to the methanogenic microcosms and sulfate-reducing microcosms, respectively. Cultures were amended with the single substrates *n*-hexadecane, ethylbenzene (both 0.1% v/v), toluene or 2-methylnaphthalene (0.5 mg of each), to investigate potential methane production rates related to the biodegradation of selected hydrocarbons. Controls without any added carbon source were incubated in parallel. Replicate cultures with 2-bromoethanesulfonate (BES; 10 mM), a specific inhibitor for methanogenic microorganisms (Scholten et al., 2000) were prepared to account for possible non-microbial methane emissions from the water or sediment samples. In sulfate-reducing microcosms, sodium azide (NaN_3_, 50 mM), a strong microbial toxin, was used to prepare metabolically inactive controls. All microcosms were incubated at 30°C in the dark and monthly sampled to assess methane and CO_2_ in the headspace as well as sulfide formation in the medium. Methane and CO_2_ production rates were calculated by linear regression of each gas increased with incubation time and expressed in μmol day^-1^ gDW^-1^ (dry weight) of sediment (Krüger et al., 2001). Methane and CO_2_ concentrations from microcosms headspace were analyzed using a methanizer-equipped gas chromatograph with flame ionization detector (GC-FID) fitted with a 6′ Hayesep D column (SRI 8610C, SRI Instruments, USA) running isothermally at 60°C, after reduction of CO_2_ to methane.

## Results and Discussion

The experimental set up of the SOFT system was too complicated for replication; however, in the following we will consider neighboring sediment layers as technical replicates, as they provide information about the plausibility of the individual data points along vertical profiles.

### Migration of Petroleum through the Sediment Core and Changes of Sediment Properties

**FIGURE 4:**
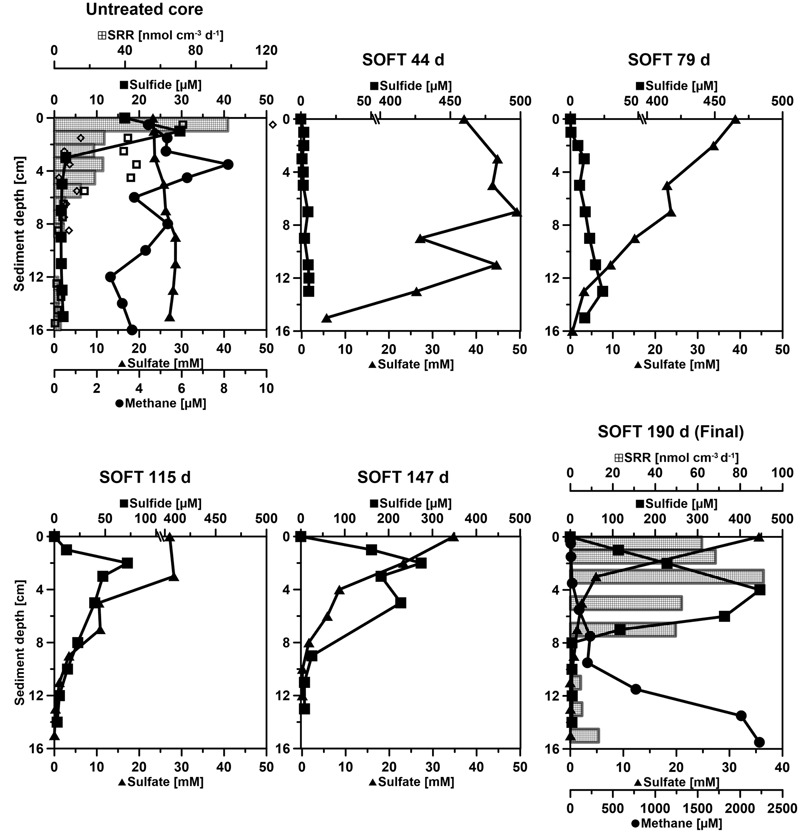
**Temporal development of biogeochemical profiles in the Caspian Sea sediment during simulated petroleum seepage.** “Untreated” shows conditions measured in a replicate core prior to the start of the SOFT experiment. “SOFT” shows condition developing over the course of 190 days in the SOFT core. Sulfate (black line with triangles), total sulfide (black line with squares), sulfate reduction rates (SRR, black checkered bars) and methane (black line with circles). Sulfide data were corrected for the shift in the electronic signal of the microsensors (between 0.5 to 1.5 mV). SRR bars (black) in the untreated core represent the average of two replicates, while the two SRR replicate values are shown as empty black squares and diamonds. In the final SOFT core (190 days) only one SRR replicate is shown. The sample at 9 cm depth was missed during radiotracer injection. Please consider the change of scale in some of the *x*-axes.

Collected sediment cores were sandy with a porosity of 0.4 throughout their length. They had an overall grayish/brown color with a black sulfidic surface layer (ca. 0.5–1 cm). Sea-grass-like plants were growing at the sediment surface. SRRs in the untreated mini cores showed highest activity in the black surface layer along with the highest sulfide accumulation in the upper 1 cm (**Figure 4**). Enhanced benthic rates of sulfate reduction and sulfide production are frequently found associated with the presence of sea grass, as the protruding plants serve as a trap for organic matter (Holmer and Nielsen, 1997; Holmer et al., 2003).

**FIGURE 5:**
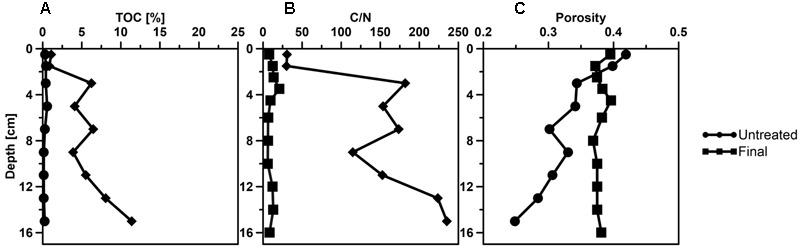
**Vertical profiles of sediment parameters (A)** total organic carbon (TOC), **(B)** C/N ratio, **(C)** porosity determined in the untreated Caspian Sea core (black line with circles) and the SOFT core (black line with squares) after 190 days of petroleum seepage.

**FIGURE 6:**
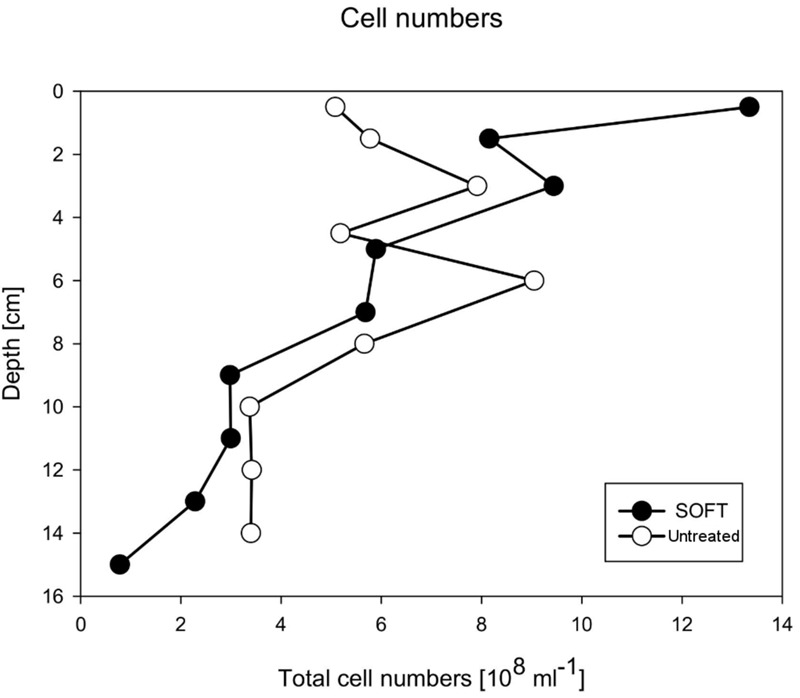
**Total cell numbers as detected by DAPI staining in the untreated and the final SOFT core**.

After the introduction of petroleum seepage, the SOFT core increasingly changed to a blackish color caused by the spreading of petroleum and sulfidic conditions. Within 1 to 2 days after the start of the seepage, oil slicks formed at the surface of the overlaying seawater generated by oil that had passed the sediment core. Petroleum droplets visibly seeped out of the sediment close to the core liner wall. High fluid flow through sediment cores can induce channelizing effects between the wall of the core liner and the sediment core, causing some fluid to move faster than the bulk volume (Steeb et al., 2014, 2015). Upon termination and slicing of the SOFT core we observed that although most of the petroleum seemed to be evenly distributed throughout the sediment, some petroleum accumulated in vein-like structures indicating such channelizing effects (Supplement 3). The migration of the bulk petroleum was indicated by the difference in the vertical distribution of TOC in the untreated and the SOFT core (**Figure 5A**). Petroleum hydrocarbons represent a form of organic enrichment of marine sediments (Bauer et al., 1988). Hence, an enrichment of TOC in the SOFT core can be interpreted as the introduction of petroleum by seepage. While a relatively low amount of TOC (0.2–0.5%) was found throughout the untreated core, TOC increased from 1 to 11.2% (**Figure 5A**) with sediment depth in the SOFT core, documenting the movement of the petroleum in the core. In accordance with the increase in TOC, the C/N ratio of the sediment drastically increased with depth up to 235 in the SOFT core as compared to a maximum of 9 in the untreated core (**Figure 5B**). Beside the variable C/N ratio of organic precursors (terrestrial or marine), petroleum becomes highly carbon enriched (C/N ∼170) during catagenesis compared to kerogen (C/N ∼40) (e.g., Hunt, 1995). Similar to our study, the C/N ratio increased with increasing oil content in sediment cores taken from an active hydrocarbon seep zone in the Coal Oil Point Field (water depth 22 m, Santa Barbara Channel, California, USA) (LaMontagne et al., 2004). While most organic-rich sediments receive organic matter input from the water column, seep sediments are mostly supplied from the subsurface through the upward flux of petroleum hydrocarbons (Reed and Kaplan, 1977; Bauer et al., 1988). As a result, some features are unique to petroleum seeps like the increase of organic carbon with sediment depth (Bauer et al., 1988). Sediment porosity in the SOFT core continuously decreased from 0.4 at the surface to 0.2 at the deepest layer (**Figure 5C**). The decrease indicates partial filling of pore spaces in the sediment with petroleum, which was not efficiently removed during the freeze-drying process of the analytical procedure for porosity determination. We assume that the pore volume of the deepest layer was probably 100% saturated with petroleum due to constant supply of petroleum from below. A porosity of 0.2 would, however, indicate that probably the more volatile fractions of the petroleum were lost during the freeze-drying process. Reduction in pore space imposes mechanical constraints on habitability of bacterial cells in sediments (Rebata-Landa and Santamarina, 2006 and references therein). Total cell counts by DAPI staining in the SOFT core revealed a decrease in cell numbers up to one-fourth below 6 cm depth and an increase in the upper half above 6 cm by a factor between 1.1 and 2.6 compared to the untreated core (**Figure 6**). The reduction in total cell number in the deeper part of the SOFT core could be the result of a decrease in available habitable pore space that was occupied by petroleum or toxicity of petroleum itself. Consequently, such mechanical constraints could limit microbial activity in a seep system, despite the presence of a rich organic carbon source.

### Development of Redox Processes in Response to Petroleum Seepage

Concentrations of dissolved electron acceptors (oxygen and sulfate) and reduced products (sulfide) in the porewater of the SOFT core indicated a vertical zonation of redox processes (**Figures 3, 4**). The zonation was in line with the natural redox ladder found in marine sediments (Jorgensen, 2006): we observed the transition from oxic to anoxic conditions, and within the anoxic sediment a separation into a top sulfate reduction and a bottom methanogenic zone.

#### Oxic Zone

Thermodynamically, oxygen is the most favorable electron donor in marine sediments (Glud, 2008) and the penetration depth (PD) of oxygen controls the depth distribution of other redox processes (Cai and Sayles, 1996). Microprofiles of oxygen concentration were taken during the SOFT experiment (**Figure 3**) from which the DOU and the PD were calculated according to Glud et al. (1994). The PD almost linearly decreased from ca. 3.8 mm on day 44 to only 2 mm after 190 days, i.e., the end of the experiment. Simultaneously, the DOU increased from 3.8 mmol m^-2^ d^-1^ on day 44 to 8.6 mmol m^-2^ d^-1^ on day 190. The total oxygen uptake (TOU) of sediment is a measure for organic carbon mineralization, as it sums up aerobic respiration as well as the oxidation of reduced chemical species produced during anaerobic respiration (Canfield et al., 1993; Glud, 2008). DOU represents the part of TOU that is dominantly mediated by microbial respiration at the seafloor and can be calculated from microsensor profiles (Glud, 2008; Boetius and Wenzhöfer, 2013). Thinning of the oxygen penetration layer indicates an increase in oxygen demand most likely as a result of microbial petroleum degradation, similar to the effect organic enrichment through pelagic carbon export has on DOU and oxygen PD in sediments (Glud et al., 1994). Likewise, sediments from cold seeps are reported to have elevated DOU rates up to two orders of magnitudes higher compared to non-seep sediments (Boetius and Wenzhöfer, 2013).

#### Sulfate-Reducing Zone

Total sulfide and sulfate concentrations steadily increased and decreased, respectively, in the sediment porewater of the SOFT core (**Figure 4**), pointing to the stimulation of sulfate-reducing bacteria (SRB). Over time, the sulfate-reducing zone moved upward, reaching its strongest development between 0 to 8 cm at the end of the incubation (190 days). At this time, sulfate penetration was limited to 8 cm (compared to 16 cm at the beginning of the experiment). While similar maxima of individual SRRs were detected in both the untreated (98.1 nmol cm^-3^ d^-1^, 0–1 cm) and the SOFT core (91 nmol cm^-3^ d^-1^, 2–4 cm) (**Figure 4**), sulfate reduction integrated over 0–16 cm doubled from 2.8 mmol SO_4_^2-^ m^-2^ day^-1^ in the untreated core to 5.7 mmol SO_4_^2-^ m^-2^ day^-1^ in the SOFT core. Marine hydrocarbon seep sediments are known to facilitate high sulfate reduction activity (Joye et al., 2004; Orcutt et al., 2010). Sulfate reduction reached some of the highest activity reported for marine sediments (244.3 mmol SO_4_^2-^ m^-2^ day^-1^ (Joye et al., 2004), which was found to be coupled mainly to hydrocarbon rather than to organic matter degradation or to the anaerobic oxidation of methane (Joye et al., 2004; Orcutt et al., 2010). In the present study from the Caspian Sea, enhanced sulfate reduction after petroleum seepage likewise pointed to the utilization of petroleum compounds by SRB. Stagars et al. (2017) discovered a high diversity of SRB in the untreated core, whose relative sequence abundance was elevated in the SOFT core at the end of the experiment. Cell numbers of hydrocarbon-degrading SRB like *Desulfobacula* and clade LCA2 increased in the sulfate-reducing zone of the SOFT core compared to the untreated core (Stagars et al., 2017). The distribution of the petroleum-degrading SRB and the increase in relative cell numbers of some petroleum-degrading groups together with elevated sulfate reduction activity in the SOFT core identified sulfate reduction as an important process in the anaerobic degradation of petroleum in Caspian Sea sediments.

#### Methanogenic Zone

**FIGURE 7:**
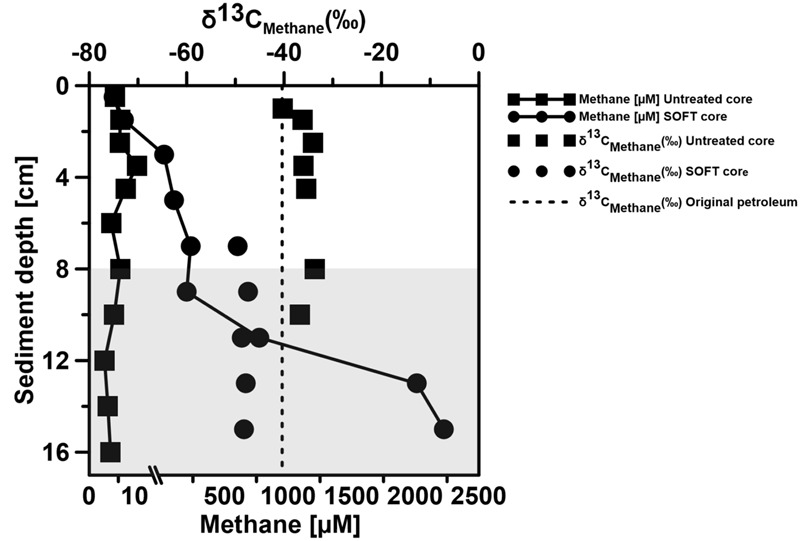
**δ^13^C of methane and methane concentration in two replicate Caspian Sea cores before (Untreated) and after the SOFT experiment (190 days, Final).** The shaded area represents the methanogenic zone of the core and the non-shaded area represents the sulfate-reducing zone. For some depths δ^13^C values are missing, because methane concentrations were too low for analyzes.

**FIGURE 8:**
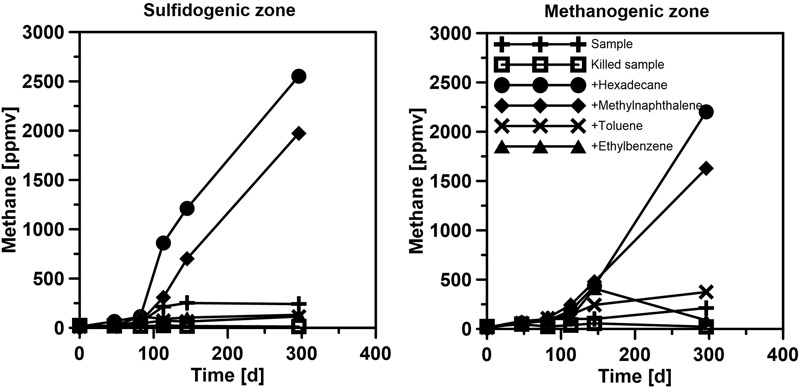
**Methane production in sulfate-free enrichment cultures in sediment samples from the sulfate-reducing (= sulfidogenic) and methanogenic zone of the SOFT core after the 190 days experiment**.

Below the sulfate reduction zone (0–8 cm), i.e., below the penetration of sulfate, the increase of methane and changes in the carbon isotopic signature of the methane (**Figure 7**) indicated the presence of a methanogenic zone. Porewater concentrations of methane increased from values between 3 and 8 μM in the untreated core up to a maximum of 2300 μM in the SOFT core below the sulfate penetration at the end of the experiment. Isotope analysis revealed a decrease in the δ^13^C signal of methane in the SOFT core (after petroleum seepage) compared to the untreated core (**Figure 7**). At 8 and 10 cm depth, the δ^13^C signal of methane decreased from -33.7 and -36.7‰, respectively, in the untreated core to -49.5 and -43.6‰, respectively, in the SOFT core. Accordingly, cell numbers of methanogenic archaea increased after petroleum seepage in the methanogenic zone (Stagars et al., 2017) and *Methanosarcina* spp. was found to be the most dominant archaeal species. The decrease in the δ^13^C signal in the SOFT core indicated a shift from a more thermogenic toward a more biogenic source of methane after the petroleum seepage (see Whiticar, 1999). The original petroleum had less than 1% methane (DEA Deutsche Erdoel AG, personal communication). Gas chromatographic headspace analyzes of original petroleum gave a concentration of 82.8 μmol/ml petroleum (Supplement 4) and a ^13^C signal of -40.3‰ for methane (**Figure 7**). A decrease in the ^13^C signal of methane to values below the supplied petroleum therefore clearly indicates microbial methane production in the SOFT core. In a study from the South Caspian Sea basin, methanogenesis was postulated for sediment cores (20 to 30 cm long, FC1 and FC2 in **Figure 2**; Jost, 2014) collected at a “non-seep” offshore site located nearby our sampling site. In FC-sediment cores, methane concentrations increased with depth and showed a typical biogenic signature of -65.5‰ indicating organic matter degradation by methanogenic archaea. Their study concluded that methanogenesis is an important process involved in the anaerobic degradation of organic matter in Caspian Sea surface sediments. In our study, the δ^13^C methane data of the untreated sediment core showed a thermogenic signature, with methane possibly sourcing from the nearby mud volcano complex (**Figure 2**). After 190 days of simulated petroleum seepage, a shift toward a more biogenic carbon isotopic signature of methane was found in the SOFT core. It can be speculated that over longer seepage time, the δ^13^C signal could have either been further shifted toward a pure biogenic signal or maintained a steady state of around -50 ‰ due to the constant inflow of fresh thermogenic petroleum (including thermogenic methane) and the partial microbial degradation/conversion of the petroleum to biogenic methane. As the amount of TOC was comparably low in the untreated sediment core (**Figure 5**), we argue that the observed methanogenesis can be attributed to petroleum degradation in the SOFT core. In order to test this hypothesis, sediment samples of the SOFT core from both the sulfate-reducing zone (0 to 8 cm) and the methanogenic zone (8 to 16 cm) were enriched in sulfate-free medium with selected petroleum hydrocarbons (hexadecane, methylnapthalene, toluene, and ethylbenzene) as sole substrates. The original crude oil used for the experiment contained 3.2 μg hexadecane per mg of crude oil (Supplement 4). Methane production was monitored over time and methanogenesis rates were calculated by linear regression (**Figure 8** and **Table 1**). Highest methanogenesis rates were observed in treatments with hexadecane (16.08 and 13.8 nmol d^-1^ml^-1^ sediment) and methylnapthalene (12.8 and 10.3 nmol d^-1^ml^-1^ sediment) in both the sulfate-reducing and methanogenic zone, respectively (**Table 1**). No statement can be made about the concentrations of methylnapthalene, toluene, and ethylbenzene, as our study focussed on *n*-alkanes only; however, it was observed that the monocyclic ethylbenzene and toluene appeared less prone to methanogenic degradation compared to the polycyclic methylnapthalene (**Table 1**). The positive response to petroleum treatment and elevated methanogenesis rates compared to the controls support the idea that methanogenesis in the SOFT core was related to the biodegradation of petroleum. It should be noted that unlike in the SOFT core, methane production was detected and highest in the enrichment cultures from the sulfate-reducing zone, suggesting that methanogens were outcompeted by SRB during the SOFT experiment in the presence of sulfate (**Figure 4**). It has been reported, while using the same substrates, that sulfate reducers outcompete methanogens at sulfate concentrations above 50 μM (Siegert et al., 2011). Therefore, in the absence of sulfate, the methanogens present in the sulfate-reducing zone could successfully use the *n*-alkanes as substrates in the sulfate-free enrichment culture studies. Several previous laboratory studies with batch cultures demonstrated methane production when offering hexadecane or petroleum indicating methanogenic degradation of these compounds (Zengler et al., 1999; Anderson and Lovley, 2000; Jones et al., 2008; Sherry et al., 2014). The SOFT system, to the best of our knowledge, is the first attempt to investigate the zonation of sulfate reducing and methanogenic processes related to degradation of petroleum hydrocarbons in a whole round sediment core under a simulated petroleum seepage.

**Table 1:** Rates of methanogenesis in enrichment cultures with sediment from the SOFT core sub-sampled at the end of the experiment.

Zone	Treatment	Methanogenesis rate (nmol d^-1^ ml^-1^ sediment)
Sulfate-reducing zone	Sample w/o additives	1.6
	Killed sample	0.0
	+ Hexadecane	16.7
	+ Ethylbenzene	0.7
	+ Methylnaphthalene	12.8
	+ Toluene	0.6
Methanogenic zone	Sample w/o additives	1.1
	Killed sample	0.0
	+ Hexadecane	13.8
	+ Ethylbenzene	0.6
	+ Methylnaphthalene	10.3
	+ Toluene	2.3

### Vertical Alteration of *n*-Alkanes and Correlation to Metabolic Processes

The present study focuses on the degradation of *n*-alkanes, because saturated hydrocarbons form the largest part of the biodegradable petroleum. Two groups of *n*-alkanes were analyzed, namely the short chain volatile *n*-alkanes (C1–C6) and selected higher *n*-alkanes (C10–C38). The higher alkanes will be henceforth categorized as lower-medium chain alkanes (C10–C14), higher-medium chain alkanes (C16–C28) and long chain alkanes (C30–C38).

**FIGURE 9:**
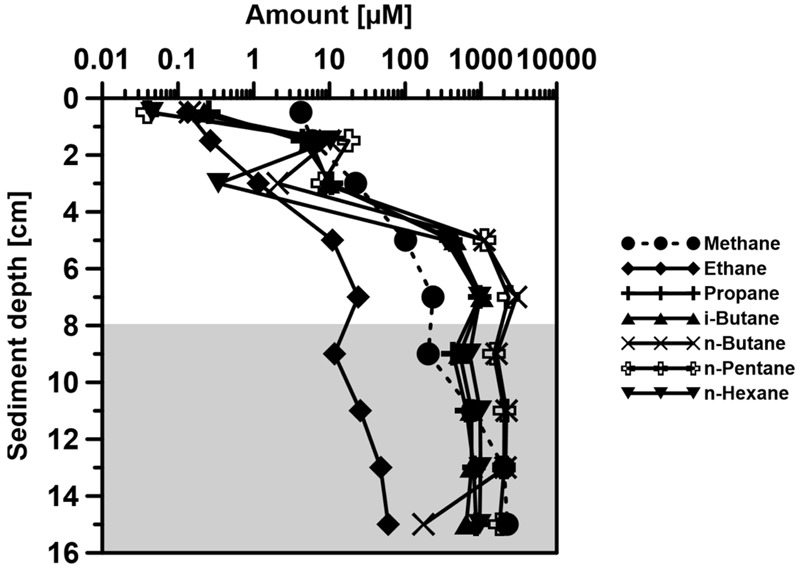
**Vertical distribution of volatile *n*-alkanes (from C1–C6: Methane, Ethane, Propane, *n*-Butane, *i*-Butane, Pentane, and Hexane) over depth in the Caspian Sea core at the end of the SOFT experiment (190 days).** The shaded area represents the methanogenic zone of the core and the non-shaded area represents the sulfate-reducing zone in the SOFT core at 190 days.

**FIGURE 10:**
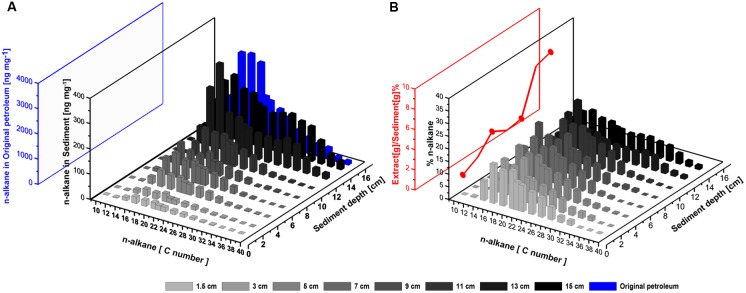
**(A)** Absolute amounts of *n*-alkanes in the Caspian Sea core after the SOFT experiment (black, ng per g sediment) and in the original, unaltered petroleum (blue, ng per mg petroleum). Surface sediment (0–1 cm) is excluded, due to possible contaminations from the settled oil slick (see text). **(B)** Relative composition of individual *n*-alkanes with respect to the sum total of all *n*-alkanes analyzed. The red line shows the ratio of the weight of petroleum extract at each depth to the respective sediment weight and represents the movement of petroleum in the SOFT core.

The short chain volatile *n*-alkanes (C1–C6) appeared to be consumed during the upward migration of the petroleum, as they almost disappeared in the upper 4 cm of the SOFT core (**Figure 9**). Methane disappeared within the sulfate-methane transition zone probably as a result of anaerobic oxidation of methane (Knittel and Boetius, 2009 and references therein). Since the top 8 cm of the core was the zone with the highest sulfate reduction activity (**Figure 4**), we postulate that sulfate reducers were mainly responsible for the degradation of the C2–C6 volatile short-chain *n*-alkanes. The relative enrichment of the higher-medium chain (C16–C28) alkanes in the upper part of the sediment further supports the selective degradation of volatile *n*-alkanes in the sulfate-reducing zone (**Figure 10B**). Additional confirmation was provided by molecular studies showing the increase in alkane-degrading SRB cell numbers in the SOFT core after petroleum flow-through (Stagars et al., 2017). We therefore, conclude that volatile *n*-alkanes escaped the methanogenic zone mostly undegraded and got degraded only after reaching the sulfate-reducing zone (8–16 cm) (**Figure 9**). Anaerobic oxidation of short-chain alkanes by SRB has been reported in marine hydrocarbon seep areas like the Gulf of Mexico and the Guaymas basin (Kniemeyer et al., 2007; Kleindienst et al., 2014).

The largest absolute amount of higher *n*-alkanes (C10–C38) was found in the deeper sediment layers, most likely as a result of the introduction of unaltered petroleum (**Figure 10A**), from where concentrations of some *n*-alkanes decreased along with the upward migration. In order to confirm that the successive decline of the C10–C38 *n*-alkanes was not just a result of non-steady vertical petroleum migration, the weight percentage of individual *n*-alkanes was calculated at each depth with respect to the sum total weight of all *n*-alkanes analyzed (**Figure 10B**). This analysis revealed a preferential degradation of lower-medium chain (<C14) and long chain (>C30) *n*-alkanes during the upward migration of petroleum among the C10–C38 *n*-alkanes. The lower-medium chain *n*-alkanes (C10–C14) were depleted throughout the core in both the sulfate-reducing and methanogenic zone (**Figures 10A,B**). The long chain *n*-alkanes (C30–C38) were mostly depleted in the methanogenic zone (**Figures 10A,B**). Preferential degradation of longer chain *n*-alkanes over shorter *n*-alkanes under anaerobic conditions have been reported before (for example, Siddique et al., 2006; Hasinger et al., 2012; Cheng et al., 2014). When offered C6–C10 *n*-alkanes, methanogens preferedly degraded *n*-alkanes in the sequence C10 > C9 > C8 > C7 > C6 (Siddique et al., 2006). It is suggested that the preferential degradation of the higher *n*-alkanes could be due to the increase in the octanol/water partition coefficient with increasing molecular weight (Siddique et al., 2006) or selective uptake of hydrocarbons across cell membranes of the hydrocarbon degrading microorganisms (Kim et al., 2002). Hasinger et al. (2012) postulate that the preferential degradation of higher *n*-alkanes under anaerobic conditions could be due to increasing biochemical energy yield with increasing chain length. The preferential degradation of longer over short chain volatile *n*-alkanes by methanogens further explains the observation that C1–C6 *n*-alkane degradation was only observed within the sulfate-reducing zone (**Figure 9**). Finally, the higher-medium range of the *n*-alkanes (C16–C28) were found to be the most persistent group of *n*-alkanes in the upward migration of the petroleum (**Figure 10B**). Further investigations are required to investigate the relative persistence of C16–C28 alkanes in comparison to the lower and higher *n*-alkanes.

A likewise vertical succession of *n*-alkane degradation was found in an oil-seep core (2 m length) collected off the coast of West Africa targeting the surface expression of a fault (Wenger and Isaksen, 2002). Thermogenic oil and gas (C1–C5 iso- and *n*-alkanes) were found throughout the core, but were essentially unaltered in the deepest layer (2 m below seafloor), while the shallower depths (from 1 m up to the seafloor) showed progressive degradation. Oil samples from Coal Oil Point seeps (Santa Barbara, CA, USA) featured a similar trend (Wardlaw et al., 2008). Here, *n*-alkanes decreased by 100% in the oil, which was seeping from the seafloor, compared to the oil of the deeper reservoir. The authors identified biodegradation as the main cause for the loss of *n*-alkanes. In the same study, it was found that physical processes like evaporation and dissolution had no significant effect on the loss of *n*-alkanes between the reservoir and the seafloor oil. Based on the observations made in the present study, i.e., the development of redox profiles, the increase in microbial activity, and the presence of hydrocarbon degraders (Stagars et al., 2017), we conclude that microbial activity led to the successive degradation of *n*-alkanes in the Caspian Sea core during the SOFT experiment.

## Conclusion

To our knowledge, this is the first study that demonstrates the use of a continuous SOFT system to investigate the response of marine surface sediment to a simulated small-scale petroleum seepage in whole round cores and facilitates comprehensive monitoring of biogeochemical parameters during the seepage simulation. We showed in coastal sediments from the Caspian Sea, taken in the vicinity of seepage structures, that microbial degradation of seeping petroleum affects the temporal and spatial distribution of redox processes and alters the composition of petroleum. After passing the sediment, petroleum was preferentially depleted in volatile *n*-alkanes (C1–C6), lower-medium chain *n*-alkanes (C10–C14) and long chain *n*-alkanes (C30–C38) over the higher-medium chain *n*-alkanes (C16–C28). Methanogenesis and sulfate reduction were identified as important processes involved in anaerobic degradation of petroleum in the selected sediments. From the depleted carbon isotopic signatures of methane following petroleum seepage, we conclude that δ^13^C signals of methane in the range of -50‰ in surface sediments probably represents a mixture of ascending thermogenic and *in situ* produced biogenic methane and could serve an indication for underlying active seepage. Using sulfide and methane as indicators, petroleum-related sulfate reduction and methanogenesis showed a response time of less than 44 and 190 days, respectively, to the induced petroleum seepage.

## Author Contributions

TT, KK, and MSc designed the study. SM and TT designed the SOFT system. SM and MSc conducted the field sampling. SM conducted the SOFT experiment, the sediment porewater and solid phase sampling, the microsensor measurements, the sulfate reduction and part of the porewater analyzes. KK conducted the cell counts. SM and PW developed and conducted the heavier *n*-alkane analyzes. MSc conducted volatile *n*-alkane analyzes and the determination of the δ^13^C of methane. MK conducted the enrichment culturing of methanogens. SM and TT wrote the manuscript with input from all co-authors.

## Conflict of Interest Statement

The authors declare that the research was conducted in the absence of any commercial or financial relationships that could be construed as a potential conflict of interest.

## Footnotes

1 seatemperature.info
