# Supplementary material for: Hydrocarbon Degradation in Caspian Sea Sediment Cores Subjected to Simulated Petroleum Seepage in a Newly Designed Sediment-Oil-Flow-Through System
Source: Front Microbiol. 2017 Apr 28;8:763. doi: 10.3389/fmicb.2017.00763 (PMC5409261; doi:10.3389/fmicb.2017.00763)
Supplement: Supplementary file 1 [file Presentation_1.pdf]

Supplementary Material

Captions

Supplement 1. Technical specification of the original SLOT (methane seepage simulation) and the modified SOFT (oil seepage simulation) system (n.a = not applied).

Supplement 2. Precision of n-alkane analyzes.

Supplement 3. Distribution of petroleum in the SOFT core after the incubation (190 d). The petroleum was relatively evenly distributed throughout the sediment but sometimes also channelized in vein-like structures (see arrows).

Supplement 4. Amount of n-alkanes in the original North Sea crude oil, which was used in the SOFT experiment. The extraction process of petroleum was repeated five times to determine the analytical precision of individual n-alkanes. The precision is represented by the standard deviation. (Values are mean, $\pm \mathrm{SD}, \mathrm{n} = 5$)

Suppl. 1

Specification	SLOT (Steeb et al. 2014)	SOFT (this study)
Methane supply from below	via advection	n.a*
Crude oil supply from below	n.a*	via advection
Sulfate supply from top	via diffusion	
Oxygen supply from top	n.a*	via diffusion (supplied by air pump)
Seawater medium delivered from top	Anoxic sulfate-rich artificial seawater medium (Widdel & Bak, 1992), salinity adapted to the respective environment	Oxic seawater prepared from sea salt (Sigma Aldrich), salinity 12 psu
Seepage medium delivered from bottom	Anoxic, sulfate-free artificial seawater medium (Widdel & Bak, 1992), salinity adapted to respective environment	n.a*
Sediment core liners	Polycarbonate core liners: gastight, total length 30 cm, inner diameter 6 cm, outer diameter 6.8 cm	
Sampling holes in core liners	3 vertical lines of 21 sampling holes (diameter 4 mm, distance between sampling holes 5.8 mm) sealed with residue-free silicon (Aquasil, Probau)	
Pore water sampling	Rhizons	
Peristaltic pumps	Medorex TL/10E, min/max pump volume 0.1/400 µL min^{-1}	
Peristaltic pump tubes	Santropen; autoclaveable, highflexible, very resistant; tubes inner diameter 0.5 mm, outer diameter 1.6 mm	
Connecting tubes	Iso-Versenic: autoclavable; very resistant; very low gas permeability; inner diameter 1 mm; outer diameter 3 mm	
Bottom sealing	PVC caps	Rubber stoppers with 2 oil channels
Top sealing	PVC cap	PVC ring covered with parafilm

*n.a = not applied

Suppl. 2

n-alkane	Standard deviation [%]<br>n=4<br>(Method precision from extraction to measurement)	Standard deviation [%]<br>n=5<br>(GC-MS precision for a standard mix of 1 ng/µL)
n-Decane (C-10)	38.9	2.2
n-Dodecane(C-12)	26.3	4.7
n-Tetradecane(C-14)	29.9	6.1
n-Hexadecane(C-16)	6.1	1.4
n-Octadecane(C-18)	3.2	1.4
n-Eicosane (C-20)	2.8	1.2
n-Heneicosane (C-21)	2.8	1.3
n-Docosane (C-22)	2.4	1.1
n-Tetracosane (C-24)	2.5	1.1
n-Hexacosane (C-26)	3.5	1.2
n-Octacosane(C-28)	3.5	1.1
n-Triacontane (C-30)	2.6	1.2
n-Dotriacontane (C-32)	3.6	1.3
n-Tetratriacontane (C-34)	4.3	1.2
n-Hexatriacontane (C-36)	4.3	1.4
n-Octatriacontane (C-38)	6.0	2.0

Suppl. 3

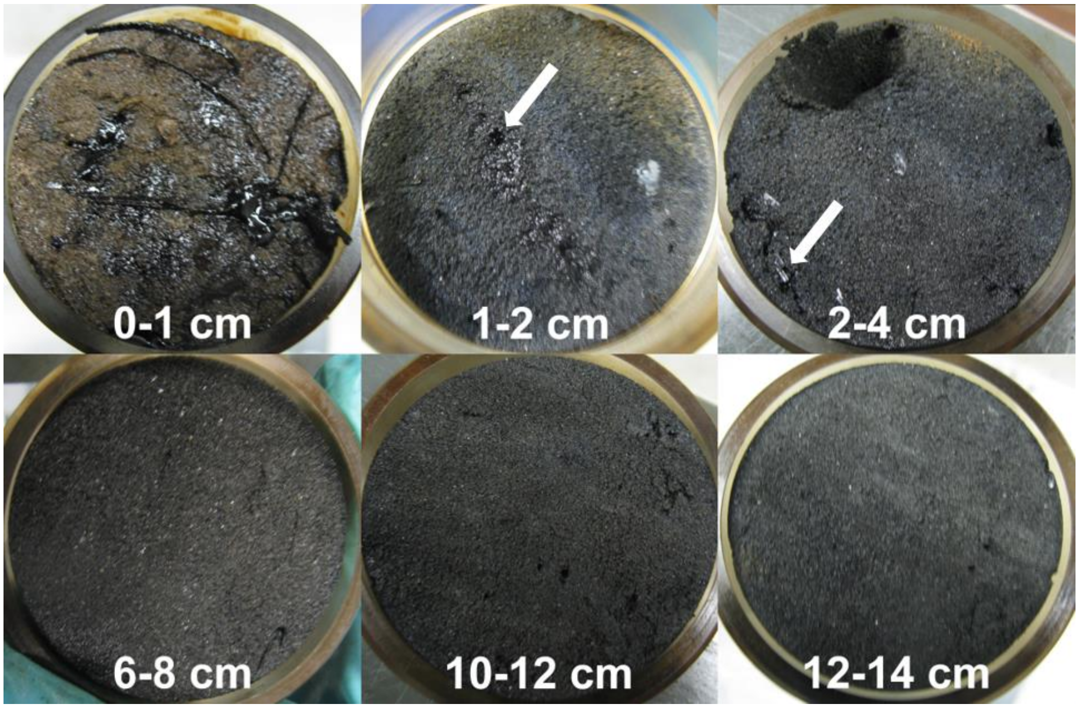

Suppl. 4

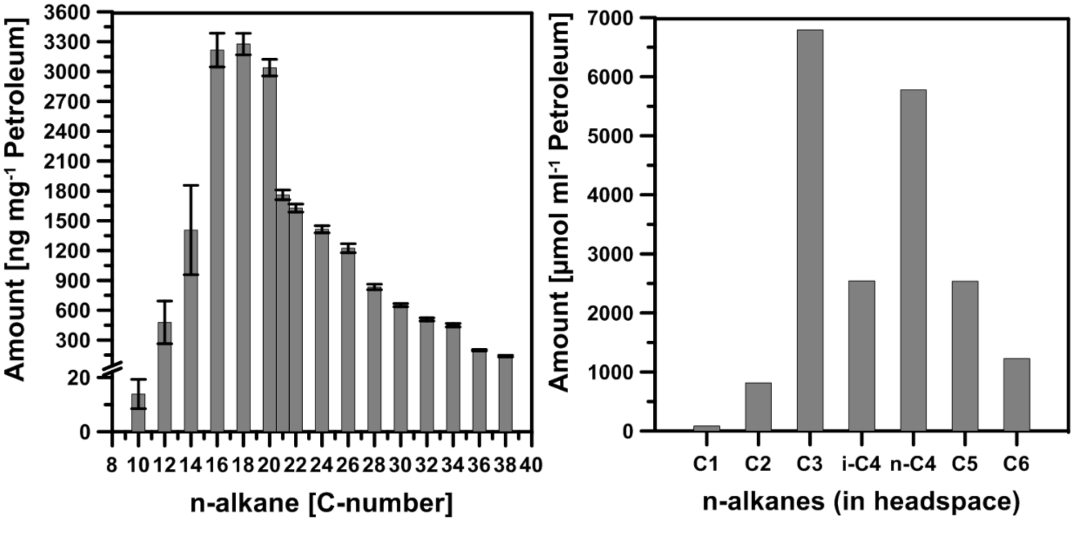

References

Widdel, F. and Bak, F. (1992). "Gram-negative mesophilic sulfate-reducing bacteria," in *The Prokaryotes*. Springer New York, 3352-3378. doi: 10.1007/978-1-4757-2191-1_21
